# Severe sepsis admitted through the emergency department to an internal medicine ward: time to antibiotic and its factors (retrospective cohort study)

**DOI:** 10.1186/2197-425X-3-S1-A222

**Published:** 2015-10-01

**Authors:** J Pires, L Ledo, G Jesus, F Ferreira, V Santos, C Madaleno, RMM Victorino, SM Fernandes

**Affiliations:** Hospital de Santa Maria, Medicina 2, Lisboa, Portugal

## Intr/Objectives

Sepsis mortality is determined by the time since hospital admission until antibiotic prescription. We aimed to evaluate the time until first antibiotic administration in patients with severe sepsis admitted directly to an Internal Medicine Ward and the factors that influence it.

## Methods

We performed a retrospective study of patients discharged from an Internal Medicine Ward with severe sepsis diagnosis between January 2011 and December 2012. Time was counted since emergency department admission. Patients on exclusive palliative care were excluded.

## Results

We identified 75 patients admitted with severe sepsis, with an average age of 75.7 ± 13 years, and an overall hospital mortality of 47%. The patients included had in average 3 severity criteria, mainly acute kidney injury (67%), altered state of consciousness (51%), hypotension (56%), and lactate above 36 mg/dL (34%). Only 31% of the patients received antibiotic treatment in the first 3 hours (mean time since hospital admission until antibiotic administration: 8.0 ± 7.9 hours). The only factor associated with timely antibiotic administration was patient direct reference to reanimation room (Odds ratio: 5.0; p < 0.01). Importantly, age, gender, and the presence of comorbidities like cardiovascular disease, cancer or dementia was not related with time until first antibiotic prescription. Moreover, the presence of classic signs of systemic inflammatory response syndrome was also not related with rapid antibiotic prescription.

## Conclusions

Understanding the factors for antibiotic delay is of utmost important to improve healthcare of severe sepsis patients. We call attention for the importance of rapid identification of these patients at the time of nurse triage in order to guarantee antibiotic administration in the first three hours. Premature nurse recognition of such patients seems to have a relevant role and therefore their training must be highlighted.

## References

[1] Angus DC, Linde-Zwirble WT, Lidicker J, Clermont G, Carcillo J, Pinsky MR (2001). Epidemiology of severe sepsis in the United States: analysis of incidence, outcome, and associated costs of care. Crit Care Med.

[2] Lefrant J-Y, Muller L, Raillard A (2010). Reduction of the severe sepsis or septic shock associated mortality by reinforcement of the recommendations bundle: a multicenter study. Ann Fr Anesth Reanim.

